# Tick-borne encephalitis vaccine effectiveness and public health impact in the Baltic countries of Estonia, Latvia, and Lithuania, 2019-2023

**DOI:** 10.1016/j.ijregi.2025.100727

**Published:** 2025-08-09

**Authors:** Frederick J. Angulo, Pingping Zhang, Milda Žygutienė, Dace Zavadska, Kerstin Aimla, Alice Kivistik, Aija Griskevica, Audrone Vadapaliene, Antra Bormane, Lisa R. Harper, Andreas Pilz, James H. Stark

**Affiliations:** 1 Global Vaccines and Anti-infectives Medical Affairs, Pfizer, Collegeville, USA; 2 Global Product Development, Pfizer, Collegevills, USA; 3 Communicable Disease Management Department, National Public Centre, Vilnius, Lithuania; 4 Department of Pediatrics, Children's Hospital, Rıga Stradins University, Riga, Latvia; 5 Department of Infectious Diseases, Tartu University Hospital, Tartu, Estonia; 6 Medical and Scientific Affairs, Pfizer, Tallin, Estonia; 7 Medical and Scientific Affairs, Pfizer, Riga, Latvia; 8 Medical and Scientific Affairs, Vilnius, Lithuania; 9 Infectious Disease Surveillance and Immunization Unit, Centre for Disease Prevention and Control of Latvia, Riga, Latvia; 10 Global Vaccines and Anti-infectives Medical Affairs, Pfizer, Vienna, Austria; 11 Global Vaccines and Anti-infectives Medical Affairs, Cambridge, USA

**Keywords:** Flavivirus, Prevention, Tick-borne diseases, Epidemiology, Public health impact

## Abstract

**Objectives:**

Tick-borne encephalitis (TBE), a potentially life-threatening disease caused by the TBE virus, is an emerging European public health threat. TBE is endemic nationwide in Estonia, Latvia, and Lithuania, which have the highest TBE incidence in Europe. TBE vaccination is recommended for all Baltic residents ≥1 year of age, but estimates of the public health impact of TBE vaccination are limited.

**Methods:**

TBE vaccination histories were determined for surveillance-reported TBE cases and for respondents from general population surveys conducted in 2019–2023. Vaccine effectiveness (VE) of ≥3 TBE doses administered in accordance with the vaccination schedule (i.e., fully vaccinated) was determined by comparing the proportion of TBE cases who were fully vaccinated (PCV) with the proportion of survey respondents fully vaccinated (PPV), using the screening method formula: VE = 1-[PCV/(1-PCV)]/[PPV/(1-PPV)].

**Results:**

There were 4,361 surveillance-reported TBE cases in the Baltic in 2019–2023; 3,806 (87.3%) were hospitalized, and 30 (0.8%) hospitalized cases died. Of 4,138 surveillance-reported TBE cases with a known TBE vaccination history, 98.3% were unvaccinated, 1.4% partially vaccinated, and 0.3% fully vaccinated. There were 89,656 participants in the general population surveys; of the 80,970 with a known TBE vaccination history, 50.1% were unvaccinated, 29.8% partially vaccinated, and 20.1% fully vaccinated. VE against TBE was 97.4% (93.0–99.0) in Estonia, 99.0% (97.7–99.5) in Latvia, and 99.6% (95% CI 98.8–99.9) in Lithuania. TBE vaccination averted an estimated 3,520 TBE cases in the Baltic in 2019–2023.

**Conclusions:**

TBE vaccination in Estonia, Latvia, and Lithuania was highly effective in preventing TBE. To prevent life-threatening TBE, TBE vaccine uptake and compliance with vaccination recommendations should be increased in the Baltic countries.

## Highlights

• Estonia, Latvia, and Lithuania have the highest incidence of tick-borne encephalitis (TBE) in Europe.
• TBE vaccination was ≥97.4% effective in preventing TBE in each of the Baltic countries.
• TBE vaccination prevented an estimated 3520 TBE cases in the Baltics in 2019-2023.
• TBE vaccine uptake should be increased in the Baltic countries.

## Introduction

Tick-borne encephalitis (TBE), a life-threatening disease caused by the TBE virus (TBEV) [1,2], is an emerging health threat in Europe, with recent geographic expansion of TBE-endemic areas [3]. European countries reported 3,516 TBE cases to the European Centre for Disease Prevention and Control (ECDC) in 2022, an incidence of 0.8 per 100,000 population per year (PPY). The TBE incidence ranged from 0.7–0.9/100,000 PPY in 2018–2022, compared with 0.4–0.6 per 100,000 PPY in 2012–2015 [4,5]. The reasons for the increased incidence are not fully understood, but it may be related to climate change, geographic expansion of the tick vector, and increased exposure to ticks [3].

**Figure 1:**
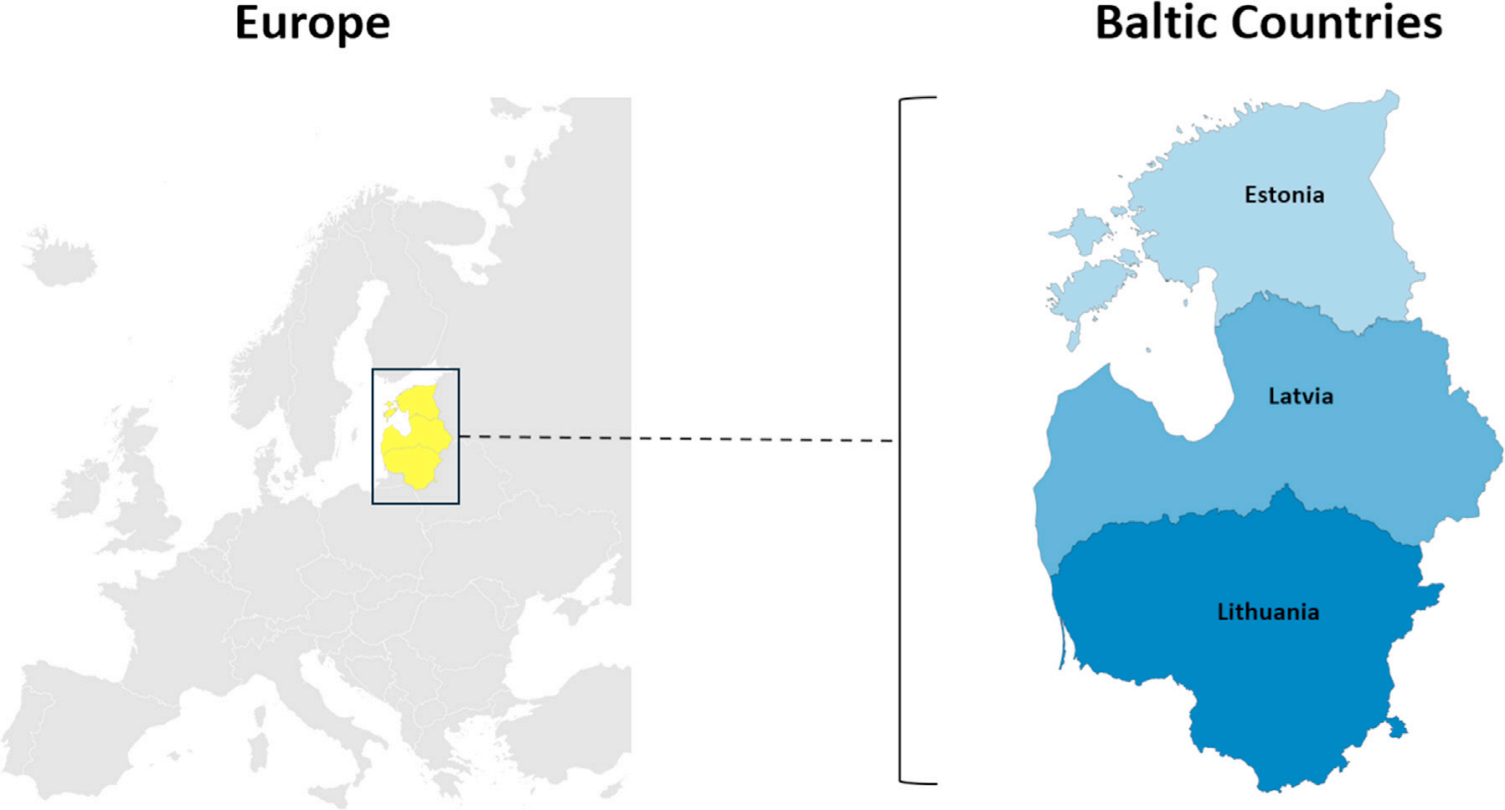
The Baltic countries (Estonia, Latvia, and Lithuania).

Twenty-five European countries have one or more TBE-endemic areas; TBE is endemic nationwide in the Baltic countries (Figure 1): Estonia (population 1.37 million), Latvia (population 1.88 million), and Lithuania (population 2.87 million) [6]. Among the countries that reported TBE cases to the ECDC in 2022, the three countries with the highest incidence of surveillance-reported TBE cases were Lithuania (12.9 per 100,000 PPY), Latvia (11.7 per 100,000 PPY), and Estonia (10.1 per 100,000 PPY) [4].

Of the five recognized TBEV subtypes, which are closely related genetically and antigenically, three subtypes (European, Siberian, and Far-Eastern) are clinically relevant in Europe [1,2]. Although the European TBEV subtype is the cause of the vast majority of TBE cases in Europe, there is evidence that the Siberian and Far-Eastern subtypes are present in the Baltic countries and Scandinavia [7]. Infection with TBEV commonly results in a febrile illness with fatigue, malaise, headache, and body pains, but asymptomatic infections may also occur [1,2]. Among symptomatic infections, initial symptoms may recede and then be followed by a second phase of neurological symptoms, ranging from mild meningitis to severe encephalitis, which can result in long-term neurological sequelae and death [1,2]. The biphasic course is more common with the European subtype [1,2]. The Far-Eastern subtype is thought to cause more severe disease and result in a higher case-fatality rate than the European subtype [1,2].

Vaccination offers the most effective protection against TBE [8]. When a TBE vaccine was introduced in Austria, TBE cases rapidly declined [9]. Two TBE vaccines, FSME IMMUN (Pfizer Europe, Brussels, Belgium) and Encepur (Bavarian Nordic, Hellerup, Denmark), are available in Europe and used in the Baltic countries. Both vaccines, which are given in a three-dose primary series, include an inactivated TBE virus of the European subtype, and FSME IMMUN has demonstrated cross-protection against the other subtypes in clinical trials [10,11]. TBE vaccination is recommended for all residents ≥1 year of age in the Baltic countries, but recent surveys indicate that less than half of the general population in each country has received the three-dose TBE vaccine primary series [12]. A major contributing reason for the low TBE vaccine uptake is that TBE vaccination is only included in the National Immunization Plan (NIP) in the Baltic countries for a narrow group of individuals (i.e., age 50–55 years in Lithuania), and therefore, most vaccine recipients must pay for the cost of vaccination. An important data gap, which may contribute to low uptake and exclusion from the NIPs, is that estimates of the effectiveness of TBE vaccines have not been published for Estonia and Lithuania, and estimates of the public health impact of TBE vaccination in the Baltic are limited. Therefore, the objectives of this study are to estimate TBE vaccine effectiveness (VE) and the public health impact of TBE vaccination in the Baltic countries, which have the highest TBE incidence in Europe.

## Methods

### Study design and population

In this observational study in the Baltic countries, vaccination histories of TBE cases reported via the national public health surveillance systems in Estonia, Latvia, and Lithuania were compared with the vaccination histories of survey respondents selected from the general population to estimate VE using the screening method. The study population included all persons in Estonia, Latvia, and Lithuania, ≥1 year of age, from 2019–2023. The study analyzed nationwide surveillance data on TBE cases routinely collected by the public health institutes and general population survey respondents; only aggregate data with no personal identifiers were utilized. Therefore, no ethical review committee approval was required when obtaining the data for the analysis.

### TBE case definition and case data

TBE is a notifiable disease in Estonia, Latvia, and Lithuania. Clinical laboratories and physicians report laboratory-diagnosed TBE cases to the public health institutes in their countries. In accordance with ECDC case definitions, a laboratory-confirmed TBEV infection is defined as a patient with anti-TBEV immunoglobulin (Ig)G and IgM antibodies in blood, seroconversion or a four-fold increase in anti-TBEV antibodies in paired serum samples, or anti-TBEV IgM antibodies in cerebrospinal fluid [13]. Summary information about the surveillance-reported TBE cases from 2019–2023, which included age and TBE vaccination history (i.e., number of doses received and date of last dose) of the cases, was obtained by request from the public health institutes. TBE vaccination histories of surveillance-reported TBE cases were collected by interview and medical record review.

### General population TBE vaccine uptake

Online household surveys, which followed standard survey procedures used by commercial marketing companies to derive national prevalence estimates, were conducted by Ipsos GmbH (Hamburg, Germany) in 2019, 2020, 2021, 2022, and 2023 to derive nationwide estimates of TBE vaccination knowledge, attitudes, and practices of individuals selected from the general population [12,14]. Participants were recruited to participate in the online survey using sampling proportions for gender, region, occupational status, and household size, based on publicly available Eurostat data (https://ec.europa.eu/eurostat). Initial participants were 18–65 years of age and, after informed consent to participate in the survey, provided information for all household members ≥1 year of age. Surveys took 5–15 minutes and gathered information on age, gender, geographic area of residence, and TBE vaccination history. Respondents consulted vaccination cards for TBE vaccination history. Survey results were weighted, using national census data, to derive national prevalence estimates. To estimate TBE vaccine uptake in the general population, survey participants were requested to consult vaccination cards for TBE vaccination histories.

### Statistical analysis

Tick-borne encephalitis incidence was estimated using surveillance-reported TBE cases and national population census estimates. The incidence of TBE in the unvaccinated population was estimated using the number of surveillance-reported TBE cases who were unvaccinated and results from the population survey to estimate the unvaccinated population. In the base case scenario, a person was considered fully vaccinated with a TBE vaccine if he or she received ≥three vaccine doses and was not late in receiving the next vaccine dose, and a person was considered partially vaccinated with a TBE vaccine if he or she (i) received fewer than three doses, (ii) received ≥three vaccine doses but was late in receiving the next vaccine dose, (iii) received ≥one vaccine dose but the number of doses received was unknown, or (iv) received ≥three vaccine doses but the dates of vaccination were unknown. Estimates of VE of ≥three TBE vaccine doses against TBE (with 95% CI) were determined by comparing the proportion of TBE cases who were fully vaccinated (PCV) and the proportion of persons in the general population surveys who were fully vaccinated (PPV) using the screening method formula VE = 1-[PCV/(1-PCV)]/[PPV/(1-PPV)], excluding persons who were partially vaccinated [15]. In the sensitivity analysis, TBE cases who received ≥one vaccine dose but the number of doses received was unknown, and TBE cases who received ≥three vaccine doses but the dates of vaccination were unknown, were assumed to be fully vaccinated instead of partially vaccinated.

The number of TBE cases averted by vaccination was estimated by calculating the TBE incidence in the unvaccinated population under a counterfactual scenario whereby persons who were vaccinated did not receive vaccination; cases averted were the difference between the number of TBE cases in the counterfactual scenario and the actual observed number of TBE cases. The number of TBE cases that would have been prevented if the entire population had been vaccinated was estimated as the product of the number of TBE cases in the counterfactual scenario and VE against TBE for persons who were vaccinated.

## Results

There were 4,361 surveillance-reported TBE cases in the Baltic countries in 2019–2023 (Estonia: 583, Latvia: 1,055, Lithuania: 2,723); an incidence of surveillance-reported TBE cases of 8.8 per 100,000 PPY in Estonia, 11.2 per 100,000 PPY in Latvia, and 19.4 per 100,000 PPY in Lithuania in 2019–2023. The number (percent) of TBE cases by age group in the Baltics in 2019–2023 was 329 (7.5%) in the 1–15 years age group, 2,618 (60.0%) in the 16–59 years age group, and 1,414 (32.4%) in the ≥60 years age group. Of the surveillance-reported TBE cases in the Baltic, 3,806 (87.2%) were hospitalized, and 30 (0.8%) of the hospitalized TBE cases died.

The range of surveillance-reported TBE cases per year in the Baltic countries in 2019–2023 was 670–1,005; 70–208 in Estonia, 176–223 in Latvia, and 365–709 in Lithuania. During the 5-year study period, the incidence of surveillance-reported TBE cases (per 100,000 PPY) ranged from 5.3 (in 2020) to 15.4 (in 2023) in Estonia, 9.4 (in 2023) to 11.9 (in 2021 and 2022) in Latvia, and 13.1 (in 2021) to 25.4 (in 2019) in Lithuania.

Of the 4,361 surveillance-reported TBE cases in the Baltic countries, 4,138 (94.9%) had a known TBE vaccination history (Table 1). Of the TBE cases with a known TBE vaccination history, 4,067 (98.3%) were unvaccinated, 58 (1.4%) were partially vaccinated, and 13 (0.3%) were fully vaccinated. With 13 fully vaccinated TBE cases during the 5-year study, the average number of TBE vaccine breakthrough cases was 2.6 per year in the Baltic. Of the 58 partially vaccinated TBE cases, 33 (56.9%) received fewer than <three doses, nine (15.5%) received ≥three doses but were late for the next dose, 14 (24.1%) received ≥one dose but the number of doses were unknown, and two (3.4%) received ≥three doses but the vaccination dates were unknown.

**Table 1:** TBE vaccination history for surveillance-reported tick-borne encephalitis cases, by country in the Baltics, 2019-2023.

	Estonia	Latvia	Lithuania	Total
*Surveillance-reported TBE cases*	n = 583 n (%)	n = 1055 n (%)	n = 2723 n (%)	n = 4361 n (%)
Known vaccination history	532 (91.3%)	1052 (99.7%)	2554 (93.8%)	4138 (94.9%)
Unknown vaccination history	51 (8.7%)	3 (0.3%)	169 (6.2%)	223 (5.1%)
*TBE cases with known vaccination history*	n = 532 n (%)	n = 1052 n (%)	n = 2554 n (%)	n = 4138 n (%)
Fully-vaccinated	4 (0.8%)	6 (0.6%)	3 (0.1%)	13 (0.3%)
Partially-vaccinated	32 (6.0%)	12 (1.1%)	14 (0.6%)	58 (1.4%)
Unvaccinated	496 (93.2%)	1034 (98.3%)	2537 (99.3%)	4067 (98.3%)
*Partially-vaccinated* *TBE cases*	n = 32 n (%)	n = 12 n (%)	n = 14 n (%)	n = 58 n (%)
<Three doses	15 (46.9)	7 (58.3)	11 (78.6)	33 (56.9)
≥Three doses late next dose	4 (12.5)	4 (33.3)	1 (7.1)	9 (15.5)
Unknown doses^a^	13 (40.6)	1 (8.3)	0	14 (24.1)
≥Three doses unknown dates^a^	0	0	2 (14.3)	2 (3.4)

a For estimating vaccine effectiveness, these cases were assumed to be partially-vaccinated in the base case scenario and fully-vaccinated in the sensitivity analysis. TBE, tick-borne encephalitis.

There were 89,656 participants in the general population surveys in the Baltic countries in 2019–2023 (Table 2), with a range of 16,509–21,016 participants per year. In the 2019–2023 surveys, there were 18,164 participants in Estonia (3273–4175 per year), 34,541 in Latvia (6255–8144), and 36,951 in Lithuania (6830–8697 per year). The number of participants in the 2019–2023 surveys by age group was 16,185 (18.0%) 1–15 years age group, 64,175 (71.6%) in the 16–59 years age group, and 9,296 (10.4%) in the ≥60 years age group. Of the participants in the 2019–2023 surveys in the Baltics, 80,970 (90.3%) had a known vaccination history. Of the participants with a known vaccination history, 40,535 (50.1%) were unvaccinated, and 40,435 (49.9%) received ≥one dose of a TBE vaccine; however, only 14,119 (34.9%) of those receiving a TBE vaccine had known dates of vaccination. Of those with known dates of vaccination, 5,677 (40.0%) were fully vaccinated, and 8,442 (60%) were partially vaccinated, indicating that the proportion of survey participants in the Baltics in 2019–2023 who were fully vaccinated was 20.1% and the proportion partially vaccinated was 29.8%. In 2019–2023, the proportion of survey respondents who were unvaccinated was 53.3% in Estonia, 38.7% in Latvia, and 59.3% in Lithuania, while the proportion of TBE cases who were fully vaccinated was 16.3% in Estonia, 21.9% in Latvia, and 18.7% in Lithuania.

**Table 2:** Tick-borne encephalitis vaccination history from general population survey, by county in the Baltics, 2019-2023.

	Estonia	Latvia	Lithuania	Total
*Among general population survey participants*	n = 18,164 n (%)	n = 34,541 n (%)	n = 36,951 n (%)	n = 89,656 n (%)
Known vaccination history	15,965 (87.9)	31,661 (91.7)	33,344 (90.2)	80,970 (90.3)
Unknown vaccination history	2199 (12.1)	2880 (8.3)	3607 (9.8)	8686 (10.7)
*Among survey participants with known vaccination history*	n = 15,965 n (%)	n = 31,661 n (%)	n = 33,344 n (%)	n = 80,970 n (%)
Vaccinated with ≥one dose	7452 (46.7)	19,422 (61.3)	13,561(40.7)	40,435 (49.9)
Unvaccinated	8513 (53.3)	12,239 (38.7)	19,783 (59.3)	40,535 (50.1)
*Among survey participants vaccinated with ≥1 dose*	n = 7452 n (%)	n = 19,422 n (%)	n = 13,561 n (%)	n = 40,435 n (%)
Known vaccinations dates	2925 (39.3)	4728 (24.3)	6466 (47.7)	14,119 (34.9)
Unknown vaccination dates	4527 (60.7)	14,694 (75.7)	7095 (52.3)	26,316(65.1)
*Among vaccinated with ≥one dose with known vaccination dates*	n = 2925 n (%)	n = 4728 n (%)	n = 6466 n (%)	n = 14,119 n (%)
Partially-vaccinated	1905 (65.1)	3038 (64.3)	3499 (54.1)	8442 (59.8)
Fully-vaccinated	1020 (34.9)	1690 (35.7)	2967 (45.9)	5677 (40.2)
*Estimates of the general population vaccine uptake*	%	%	%	%
Fully-vaccinated^a^	16.3	21.9	18.7	20.1
Partially-vaccinated^b^	30.4	39.4	22.0	29.8
Unvaccinated	53.3	38.7	59.3	50.1

a Derived by multiplying the proportion of respondents vaccinated with ≥one dose by the proportion of respondents vaccinated with ≥one dose with known date of vaccination who were fully-vaccinated. ^b^Derived by multiplying the proportion of respondents vaccinated with ≥one dose by the proportion of respondents vaccinated with ≥one dose with known date of vaccination who were partially-vaccinated.

Using results from surveillance and the population surveys, the incidence of surveillance-reported TBE in the unvaccinated population from 2019–2023 was 14.1 per 100,000 PPY in Estonia, 28.5 per 100,000 in Latvia, and 30.5 per 100,000 in Lithuania. In the base case scenario, the VE of ≥three TBE vaccine doses administered according to the vaccination schedule in individuals ≥1 year-of-age against TBE in 2019–2023 was 97.4% (95% CI 93.0–99.0) in Estonia, 99.0% (95% CI 97.7–99.5) in Latvia, and 99.6% (95% CI 98.8–-99.9) in Lithuania (Table 3). When stratified by age groups by country, VE in the 1–15 years age group was 90.8% (95% CI 62.5–97.7) in Estonia, 93.5% (95% CI 73.5–98.4) in Latvia, and 100% (95% CI undefined) in Lithuania (Supplemental Table). The VE in the 16–59 years age group ranged from 97.7% (95% CI 90.6–99.4) in Estonia to 99.6% (CI 98.5–99.9) in Lithuania, and in the ≥60 years age group ranged from 99.4% (95% CI 95.8–99.9) in Lithuania to 100% (95% CI undefined) in Estonia and Latvia.

**Table 3:** VE with 95% CIs of three or more doses of a TBE vaccine administered according to the vaccination schedule in individuals ≥1 year-of-age, by country in the Baltics, 2019-2023.

	Total TBE cases	Unvaccinated TBE cases	Base case scenario		Sensitivity analysis	
			Fully-vaccinated TBE cases	VE (95% CI)	Fully-vaccinated TBE cases	VE (95% CI)
Estonia	583	496	4	97.4 (93.0-99.0)	17	88.8 (81.9-93.1)
Latvia	1,055	1,034	6	99.0 (97.7-99.5)	7	98.8 (97.5-99.4)
Lithuania	2,723	2,537	3	99.6 (98.8-99.9)	5	99.4 (98,5-99.7)

VE, vaccine effectiveness; CI, confidence interval; TBE, tick-borne encephalitis.

In the sensitivity analysis, the 14 cases (13 in Estonia and one in Latvia) that received ≥one dose but for whom the number of doses were unknown, and the two cases in Lithuania that received ≥three doses but for whom the vaccination dates were unknown, were assumed to be fully vaccinated rather than partially vaccinated, Under this assumption, the VE of ≥three TBE vaccine doses administered according to the vaccination schedule in individuals ≥1 year of age against TBE in 2019–2023 was 88.8% (95% CI 81.9–93.1) in Estonia, 98,8% (95% CI 97.5–99.4) in Latvia, and 99.4% (95% CI 98.5–99.7) in Lithuania.

In 2019-2023, TBE vaccination averted 3,520 TBE cases in the Baltic countries: 350 TBE cases in Estonia, 1,620 TBE cases in Latvia, and 1,550 TBE cases in Lithuania. If the entire population in the Baltics had been fully vaccinated with a TBE vaccine in 2019–2023, TBE vaccination would have prevented 7,817 TBE cases in the Baltics: 908 TBE cases in Estonia, 2,650 TBE cases in Latvia, and 4,259 TBE cases in Lithuania.

## Discussion

Our analysis of surveillance-reported TBE cases and surveys of the general population in the Baltic countries found that TBE vaccination in 2019–2023 resulted in a substantial beneficial public health impact in Estonia, Latvia, and Lithuania, countries with the highest TBE incidence in Europe. Our study also demonstrated that receipt of ≥three TBE vaccine doses administered in accordance with the vaccination schedule was >97% effective in preventing TBE in each of the Baltic countries. Furthermore, during the 5-year study period, TBE vaccination prevented >3,500 TBE cases in the Baltic. Since 87% of the surveillance-reported TBE cases during the study period were hospitalized, the prevention of these TBE cases represents an important beneficial impact for the healthcare systems in these countries. We also demonstrate that TBE vaccines were highly effective in preventing TBE in children, a population in which TBEV infection can result in important cognitive declines [16,17], and highlight the need for further availability of TBE vaccines for children in the Baltic. These findings, which are the first to demonstrate the public health impact of TBE vaccination and TBE VE in Estonia and Lithuania, are consistent with studies that have demonstrated high TBE VE and beneficial public health impact in Austria, the Czech Republic, Germany, Sweden, and Switzerland [17], [18], [19].

Despite the high effectiveness of TBE vaccines in the Baltic countries, there were 4,361 surveillance-reported TBE cases in the three countries during the study period. In our study, 98.3% of the TBE cases were unvaccinated despite TBE, a potentially disabling and life-threatening disease, being endemic nationwide in Estonia, Latvia, and Lithuania, and TBE vaccination being recommended for all Baltic residents >1 year of age. Results from our population surveys demonstrate that less than half of the population in the Baltic countries has received ≥one doses of a TBE vaccine, and only 20% of the population is fully vaccinated against TBE. The high risk of TBE in the unvaccinated population in the Baltic is demonstrated by our study’s high estimated TBE incidence in the unvaccinated populations in Estonia (14.1 per 100,000 PPY), Latvia (28.5 per 100,000 PPY), and Lithuania (30.5 per 100,000 PPY). Importantly, without TBE vaccination, the TBE disease burden in the Baltic would have been even higher. Furthermore, if the entire population had been fully vaccinated against TBE during the study period, TBE vaccination would have prevented 7,817 TBE cases. These results highlight that TBE vaccine uptake in the Baltic is suboptimal and contributes to the high TBE incidence in these countries, and that enhanced efforts are needed to increase TBE vaccine uptake in Estonia, Latvia, and Lithuania.

Given that the cost of vaccination can be an important barrier to electing to receive TBE vaccination, consideration should be given to either the inclusion of TBE vaccination in the NIPs of the Baltic countries or the establishment of other approaches for government support for the cost of TBE vaccination. Establishing government support for the cost of TBE vaccination, such as including TBE vaccination in the NIPs, rather than requiring payment for vaccination by recipients, would likely markedly increase TBE vaccine uptake.

A limitation of this study is that the VE estimates were derived via the screening method, which compared the vaccine history of patients with TBE with that of survey responders selected from the general population. Although the general population surveys were conducted using standard population survey techniques, the potential for selection bias remains. In particular, individuals who were TBE vaccinated may have been more likely to participate in the population survey than individuals who were unvaccinated. If this occurred, the TBE vaccine uptake of the general population would be overestimated, and TBE VE would be underestimated. Also, the TBE vaccination history could not be determined for 5.1% of TBE cases and 10.7% of survey participants; we do not know if individuals with unknown TBE vaccination histories were more or less likely to have received a TBE vaccine, or if this differed between TBE cases and survey participants. Therefore, we conducted a sensitivity analysis, which demonstrated high VE even in the unlikely event that all TBE cases with an unknown TBE vaccination history were fully vaccinated against TBE. Differential recall of vaccine history by the survey participants and the TBE cases may be present, since different approaches were used to ascertain the vaccine history of TBE cases and of the surveyed general population. This could result in misclassification bias. Although the direction of the selection and misclassification bias in the screening method approach is unknown, two recent studies estimated VE using both a matched case-control analysis and the screening method and found lower, and thereby more conservative, VE estimates using the screening method, suggesting that the bias using the screening method may be towards the null, resulting in lower VE estimates [20,21].

In conclusion, we found that TBE vaccines were highly effective in preventing TBE in Estonia, Latvia, and Lithuania, but TBE vaccine uptake is low; fewer than half the population in the Baltic countries is fully vaccinated against TBE. To prevent life-threatening TBE, TBE vaccine uptake and compliance with vaccine recommendations should be increased in Estonia, Latvia, and Lithuania. Furthermore, given the clinical severity of TBE, the fact that the Baltic countries have the highest TBE incidence in Europe, and that TBE vaccination is highly effective at preventing TBE, consideration should be given to government support for the costs of TBE vaccination in Estonia, Latvia, and Lithuania.

## Declaration of competing interest

FJA, PZ, AK, AG, AV, LRH, AP, and JHS are employees of Pfizer Inc. and hold stock and stock options in Pfizer Inc. All other co-authors declare no conflicts of interest.

## Appendix Supplementary materials

Image, application 1
