# Supplementary material for: Tick-borne encephalitis vaccine effectiveness and public health impact in the Baltic countries of Estonia, Latvia, and Lithuania, 2019-2023
Source: IJID Reg. 2025 Aug 9;16:100727. doi: 10.1016/j.ijregi.2025.100727 (PMC12452677; doi:10.1016/j.ijregi.2025.100727)
Supplement: Supplementary file 1 [file mmc1.docx]

Supplemental table. Vaccine effectiveness with 95% confidence intervals of three or more doses of a tick-borne encephalitis vaccine administered according to the vaccination schedule in individuals ≥1 year-of-age, by age group and country in the Baltics, 2019-2023.

| Age group in years | Estonia | | | | Latvia | | | | | Lithuania | | | | |
| --- | --- | --- | --- | --- | --- | --- | --- | --- | --- | --- | --- | --- | --- | --- |
|  | Total TBE cases | Unvac-cinated cases | Fully-  vaccinated cases | VE  (95% CI) | | Total TBE cases | Unvac-cinated cases | Fully-  vaccinated cases | VE  (95% CI) | | Total TBE cases | Unvac-cinated cases | Fully-  vaccinated cases | VE  (95% CI) |
| 1-15 | 96 | 78 | 2 | 90.8 (62.5-97.7) | | 68 | 63 | 2 | 93.5 (73.5-98.4) | | 165 | 164 | 0 | 100 (undefined) |
| 16-59 | 289 | 252 | 2 | 97.7 (90.6-99.4) | | 670 | 659 | 4 | 99.0 (97.4-99.6) | | 1659 | 1521 | 2 | 99.6 (98.5-99.9) |
| ≥60 | 198 | 166 | 0 | 100 (undefined) | | 317 | 312 | 0 | 100 (undefined) | | 899 | 852 | 1 | 99.4 (95.8-99.9) |

CI, confidence interval; TBE, tick-borne encephalitis; VE, vaccine effectiveness.
